# CoryneRegNet: An ontology-based data warehouse of corynebacterial transcription factors and regulatory networks

**DOI:** 10.1186/1471-2164-7-24

**Published:** 2006-02-14

**Authors:** Jan Baumbach, Karina Brinkrolf, Lisa F Czaja, Sven Rahmann, Andreas Tauch

**Affiliations:** 1 International NRW Graduate School in Bioinformatics and Genome Research, Centrum für Biotechnologie, Universität Bielefeld, Universitätsstraße 25, D-33615 Bielefeld, Germany; 2 Algorithms and Statistics for Systems Biology Group, Genome Informatics, Technische Fakultät, Universität Bielefeld, Universitätsstraße 25, D-33615 Bielefeld, Germany; 3 Institut für Genomforschung, Centrum für Biotechnologie, Universität Bielefeld, Universitätsstraße 25, D-33615 Bielefeld, Germany

## Abstract

**Background:**

The application of DNA microarray technology in post-genomic analysis of bacterial genome sequences has allowed the generation of huge amounts of data related to regulatory networks. This data along with literature-derived knowledge on regulation of gene expression has opened the way for genome-wide reconstruction of transcriptional regulatory networks. These large-scale reconstructions can be converted into in silico models of bacterial cells that allow a systematic analysis of network behavior in response to changing environmental conditions.

**Description:**

CoryneRegNet was designed to facilitate the genome-wide reconstruction of transcriptional regulatory networks of corynebacteria relevant in biotechnology and human medicine. During the import and integration process of data derived from experimental studies or literature knowledge CoryneRegNet generates links to genome annotations, to identified transcription factors and to the corresponding cis-regulatory elements. CoryneRegNet is based on a multi-layered, hierarchical and modular concept of transcriptional regulation and was implemented by using the relational database management system MySQL and an ontology-based data structure. Reconstructed regulatory networks can be visualized by using the yFiles JAVA graph library. As an application example of CoryneRegNet, we have reconstructed the global transcriptional regulation of a cellular module involved in SOS and stress response of corynebacteria.

**Conclusion:**

CoryneRegNet is an ontology-based data warehouse that allows a pertinent data management of regulatory interactions along with the genome-scale reconstruction of transcriptional regulatory networks. These models can further be combined with metabolic networks to build integrated models of cellular function including both metabolism and its transcriptional regulation.

## Background

Microorganisms continuously have to handle changing environmental conditions to maintain their functional homeostasis and to overcome stress situations with detrimental consequences for growth and survival [1]. Therefore, they evolved mechanisms to sense alterations within their environmental surroundings and developed molecular strategies co-ordinated by complex transcriptional regulatory networks to manage unfavourable conditions. The complexity of such regulatory networks results from the interaction of numerous transcription units consisting of a transcription factor and a defined set of regulated target genes [2]. The most important components of these units are apparently the DNA-binding transcription factors. They are responsible for sensing environmental and intracellular signals to control cellular reproduction and growth [3], and they include a DNA-binding domain that possesses a secondary structure to recognize the operator sequences of regulated genes [4]. Depending on the growth conditions of a bacterial cell certain fractions of the total set of transcription factors are operating [5]. Some of them only control the expression of a single gene whereas others organize the activation or repression of numerous target genes [2].

The availability of whole genome sequences provides the opportunity to define the total set of DNA-binding transcription factors of an organism [6,7]. This is a first step not only in understanding the regulatory complexity of a certain bacterial cell but also for reconstructing the global connectivity of a regulatory network to theoretically describe and deduce gene expression pattern of a microorganism [8]. From a set of complete genome sequences it has been deduced that large genomes include more transcription factors per gene than small genomes [9]. The increase of genomic complexity is thus associated with a more complex regulation of gene expression since the additional genetic information has to be integrated into the existing regulatory network basically operating in a bacterial cell. The transcriptional regulatory network of *Escherichia coli *so far is one of the best characterized regulatory systems of a single cell. The total number of about 320 transcriptional regulators of *E. coli *K-12 were classified into eight distinct regulatory modules with defined physiological functions [5]. Additional bioinformatics studies suggested a hierarchical and modular structure of the regulatory network, excluding circular feedback loops on transcriptional level for this organism [10].

The genus *Corynebacterium *comprises a number of human pathogens, like *Corynebacterium diphtheriae *and *Corynebacterium jeikeium*, as well as the non-pathogenic soil bacteria *Corynebacterium glutamicum *and *Corynebacterium efficiens *that are widely used in biotechnological production processes of food and feed additives [11,12]. Because of their relevance in biotechnology and medicine the genome sequences of *C. glutamicum *ATCC 13032, *C. efficiens *YS-314, *C. diphtheriae *NCTC 13129, and *C. jeikeium *K411 have recently been determined [13-16]. First comparative analysis revealed a high-level conservation of orthologous genes in these genome sequences, indicating that the corynebacterial species have rarely undergone genome rearrangements and thus largely retained their ancestral genome structure [17]. An initial step in understanding the transcriptional regulatory machinery of corynebacteria was the bioinformatics identification of the encoded transcription factors [7]. A collection of 127 DNA-binding transcription factors was detected in the genome sequence of *C. glutamicum*, whereas 103 regulators were identified in *C. efficiens*, 63 in *C. diphtheriae *and 55 in *C. jeikeium*. The relation between these numbers agrees well with the assumption that the quantity of transcription factors of an organism is correlated to the genome size and the environmental surrounding a bacterial cell is exposed to [9]. Accordingly, the physiological versatility of *C. glutamicum *results in a considerably higher number of transcriptional regulators, and in consequence in a more complex regulatory network by integrating and co-ordinating additional regulatory subnetworks. According to amino acid comparisons and protein structure predictions the repertoire of DNA-binding transcription factors of *C. glutamicum*, *C. efficiens*, *C. diphtheriae*, and *C. jeikeium *were further on divided into 25 families of regulatory proteins. A common set of only 28 regulators was encoded by all of the four genome sequences and thus presumably includes the core set of DNA-binding transcription factors of these bacteria [7]. Despite the progress in bioinformatics prediction of transcription factors, the reconstruction of regulatory networks is generally hindered by the relatively low level of evolutionary conservation of other molecular network components, for instance of the cognate operator sequence of a DNA-binding transcription factor. However, developments in DNA microarray technology have allowed the generation of genome-wide data sets characterizing experimentally the regulatory networks of corynebacteria [18-20].

The ambition of our current post-genomic approaches is to decipher and reconstruct the transcriptional regulatory network of *C. glutamicum*. Here, we propose a hierarchical and modular scheme of the regulatory network, separating the repertoire of DNA-binding transcription factors into five well-defined and functionally distinct modules with respect to the physiological role of the regulated target genes. This biological concept was applied to design and implement the ontology-based data warehouse CoryneRegNet that provides a solid basis for further regulatory network studies in the field of systems biology. As an application example of CoryneRegNet we reconstructed and visualized the functional module "SOS and stress response" of *C. glutamicum*, revealing a multi-layered, hierarchical and modular structure of the respective transcriptional regulatory interactions.

## Construction and content

### The biological concept of CoryneRegNet

**Figure 1:**
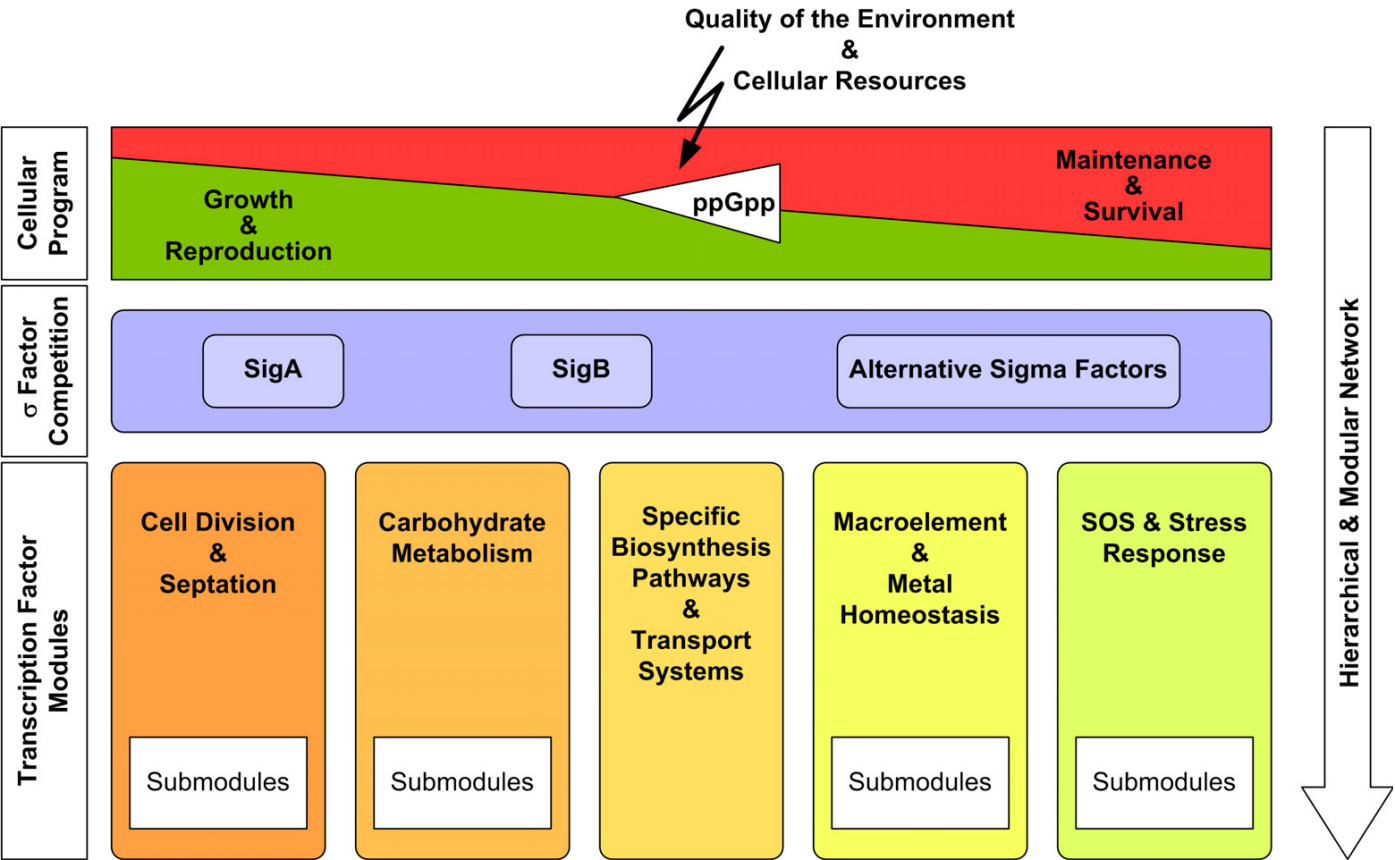
The biological concept of CoryneRegNet. The model presents the hierarchical and modular network structure of transcriptional regulatory interactions in *C. glutamicum*. It consists of five distinct transcription factor modules and a module containing the main and alternative sigma factors involved in differential gene expression by sigma factor competition [7]. A top level regulator is the hyperphosphorylated guanosine nucleotide ppGpp, involved in sensing the quality of the environment and the cellular resources [7]. The amount of ppGpp determines the cellular program and the role of sigma factor competition in global regulation of gene expression.

As a prerequisite for database design a biological concept of global transcriptional regulation of *C. glutamicum *was developed that later on was converted into a generalized and ontology-based data structure. In a first step, the total set of 127 DNA-binding transcription factors identified in the genome sequence of *C. glutamicum *[7] was classified into five functionally distinct modules (Figure 1). Using these categories along with the genome annotation, 68.3% of the transcription factors were grouped into the modules according to their proposed or evolutionary conserved function [7]. Transcription factors that did not fit into a module remained unclassified. Consequently, each transcription factor module includes several regulatory units composed of a transcription factor and a specific number of regulated genes. These units might be linked to form physiologically distinct submodules within the functional classes that might in turn assemble into a larger network including regulatory interactions between different transcription factor modules. A higher level of regulation of gene expression is represented by the seven sigma factors of *C. glutamicum *[14] that might exert their function in differential gene expression by the molecular mechanism of sigma factor competition [21,22]. As subunits of the RNA polymerase holoenzyme they recognize specific promoter sequences and alter the transcriptional profile of a cell in response to changing environmental conditions. Thereby sigma factors can influence the expression of certain target genes as well as of genes encoding transcription factors or even sigma factors. When adapting information from an *E. coli *model on hierarchical regulation of gene expression, an important environmental signal input into transcriptional regulation of *C. glutamicum *occurs at the top level of the regulatory cascade that comprises the cellular programs "growth and reproduction" and "maintenance and survival" (Figure 1). Which of these programs dominates the actual physiological state of a cell is dependent on environmental conditions and the cellular resources and is inversely correlated with the cellular amount of the hyperphosphorylated guanosine nucleotide, ppGpp [21,23]. An accumulation of ppGpp occurs for instance in response to amino acid depletion triggering the so-called stringent response, but varying amounts of ppGpp were also observed in consequence to other environmental stresses [23]. In *C. glutamicum*, an increase in ppGpp synthesis is inversely correlated with growth rate and energy production of the cell [24,25]. However, the role of ppGpp as global regulator of gene expression in *C. glutamicum *awaits experimental verification. Respective studies in *E. coli *revealed that ppGpp directly intervenes with sigma factor competition by binding to the RNA polymerase core enzyme [23]. Consequently, this biological concept displays a hierarchical and modular structure composed of (i) the components determining the cellular program, (ii) the components of the sigma factor module involved in sigma factor competition and (iii) the five defined functional modules containing the complete repertoire of DNA-binding transcription factors predicted for *C. glutamicum *(Figure 1).

### The database concept of CoryneRegNet

**Figure 2:**
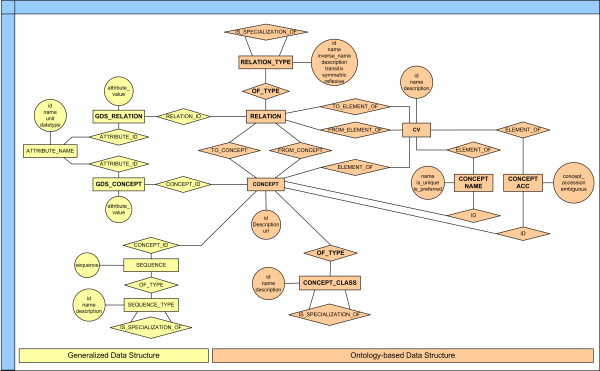
Entity Relationship (ER) diagram representing the data structure used for the construction of CoryneRegNet. The ER was implemented in the DBMS MySQL and is divided into two main parts: the generalized data structure (*GDS*) and the ontology-based data structure. Rectangles represent entities, rhombi represent relations between two entities and circles represent attributes of entities. The entities *Concept *and *Relation*, which are the main components of the ontology-based data structure, are located in the center of the ER diagram. They store all essential data on genes, proteins and functional modules as well as every linkage between them. They are typed (*Concept_class *and *Relation_type*) and link to the controlled vocabulary (*CV*) they have been extracted from. Furthermore, they link to their generalized attributes (*GDS_relation *and *GDS_concept*) and to associated sequences (entity *Sequence*). Alternative names and accessions are stored in the tables of the entities *Concept_name *and *Concept_accession*.

Generally, any kind of biological data can be considered as an ontology, which consists of concepts that are linked through relations. Accordingly, the goal was to integrate heterogenous data related to transcriptional regulation into a database in such a way that they fit into a single ontology-based data structure. In principle, technical and semantic data integration can be performed during data import. If a mechanism exists that ensures the correct semantics of the relations, then different data sources from different levels of biological hierarchy can be integrated into the same database scheme [26]. The data that have to be imported can be regarded as a set of structured and named concepts and the respective data sources are thus so-called controlled vocabularies (CVs). In CoryneRegNet, the data are first imported into a data repository, thereby creating a dataset concept for each biological entity, for instance genes, proteins or transcription factors, and a dataset relation for any connection between two concepts. Figure 2 shows the Entity Relationship diagram of CoryneRegNet, which was implemented in MySQL and which is similar to other ontological data structures, such as the ONDEX (ONtological inDEXing) data structure [26]. The most important entities located in the center of the diagram are *Concept *and *Relation *(Figure 2). These two entities are connected by the relations *From_concept *and *To_concept*. *Relations *and *Concepts *are typed and accordingly there are the entities *Concept_class *and *Relation_type *that reference themselves by the relationship *Is_specialization_of*. For instance *Transcription factor *is a specialization of *Protein *or the relation *Activates *is a specialization of *Binds_to*. Additionally, every concept and every relation links to the CV it has been extracted from. In particular, there are references such as *Element_of*, *From_element_of *and *To_element_of *that link the concept item and relation item to the entity *CV*, respectively (Figure 2). Furthermore, accessions are unique identifiers that unambiguously determine a concept but solely within a specific CV. *Concepts *might have different names and accessions in different databases. This information is stored in tables for the entities *Concept_name *and *Concept_accession*. Moreover, concepts and relations might have attached values, for instance genes have start and stop positions within the genome sequence, whereas transcription factors can be associated with position weight matrices deduced from the cognate DNA-binding sites. Thus, it is necessary to have also a generalized data structure, the entity *GDS*, to store concept or relation specific values in a generalized way (Figure 2).

**Figure 3:**
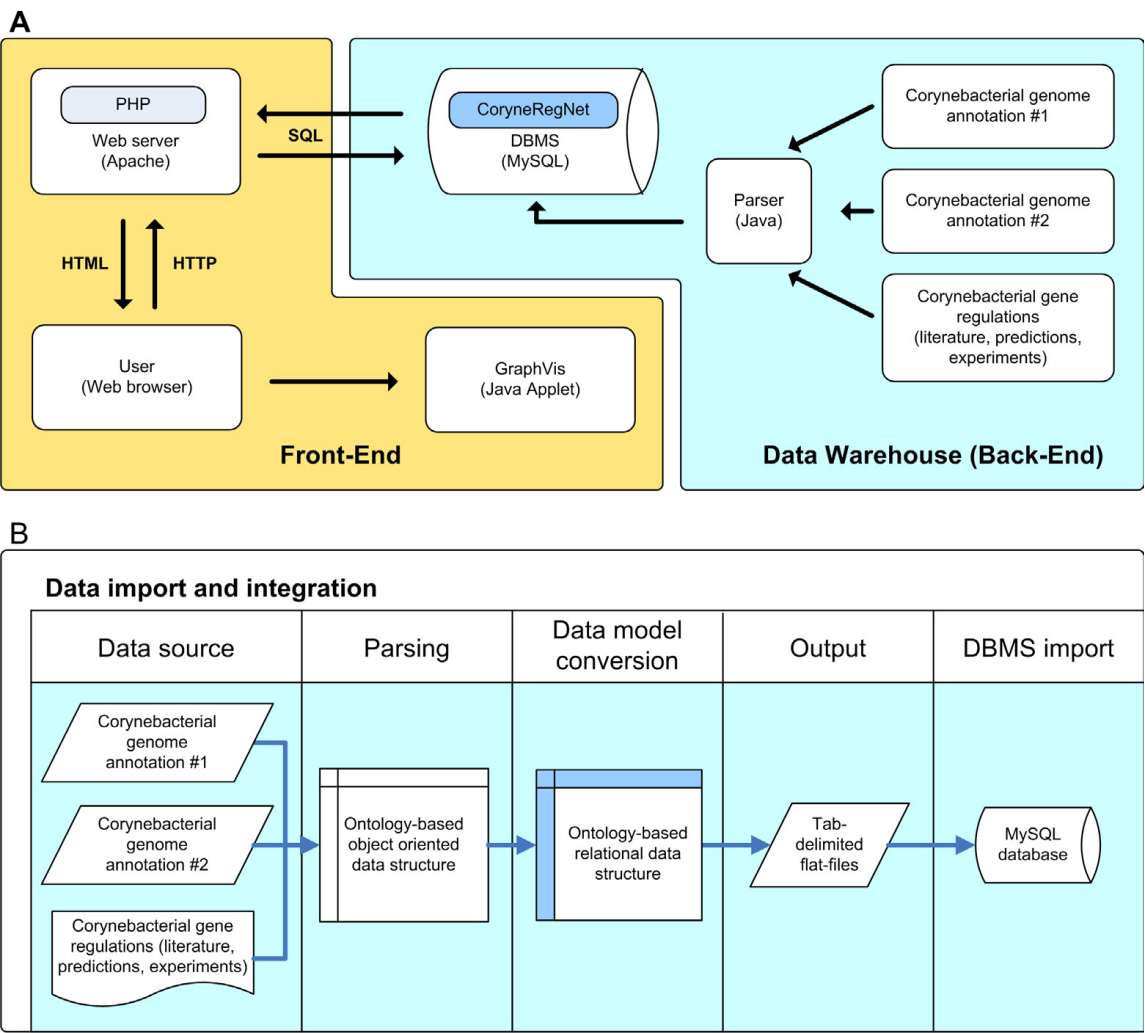
Overview of the CoryneRegNet system (A) and the data import and integration process (B). The front-end consists of an Apache Web server, which queries the CoryneRegNet database back-end and constructs the browser-sided user interface and GraphVis, a Java applet that visualizes a queried result. The back-end is a data warehousing system that cross-links two corynebacterial genome annotations together with gene regulations and integrates the respective data into a single database. Data import and integration is illustrated in more detail in (B).

According to this principle, CoryneRegNet is designed as a web-based software environment (Figure 3A) that is publicly available. At the front-end, the scripting language PHP 4.3.2  is used for the development of a user interface. CoryneRegNet runs on an Apache HTTP server 2.0.49  and queries the open source database management system MySQL 4.1.9 , which is used for data storage. The program enabling data import and data integration is implemented in Java 1.4.2  as is the graph-based network visualization tool GraphVis, which uses an academic licence version of the yFiles JAVA graph library [27]. The entire system was developed and runs on servers configured with the operating system Solaris 9/SunOS 5.9.

For the reconstruction of corynebacterial regulatory networks, the complete genome sequence of *C. glutamicum *along with the genome annotation [14] was downloaded from NCBI [28] in GenBank format and imported into CoryneRegNet. Subsequently, the gene identifiers were mapped to a second *C. glutamicum *genome sequence and annotation [29] to enable scientists working with either of the two annotations the efficient usage of CoryneRegNet. Furthermore, biological data relevant to transcriptional regulations were imported into the database as derived from literature knowledge (included in the database as PubMed link) [30], computer predictions [7] or experimental studies [19,20] (Figure 3B). The data import process was realized by running a parser that was implemented in Java. The parser software additionally integrates the imported data into a single ontology-based data structure and converts it into a relational data model (Figure 2). The output are tab-delimited flat-files that in turn are input files for the MySQL built-in import procedure and finally used to fill the CoryneRegNet database (Figure 3B).

## Utility and discussion

### The user interface of CoryneRegNet

**Figure 4:**
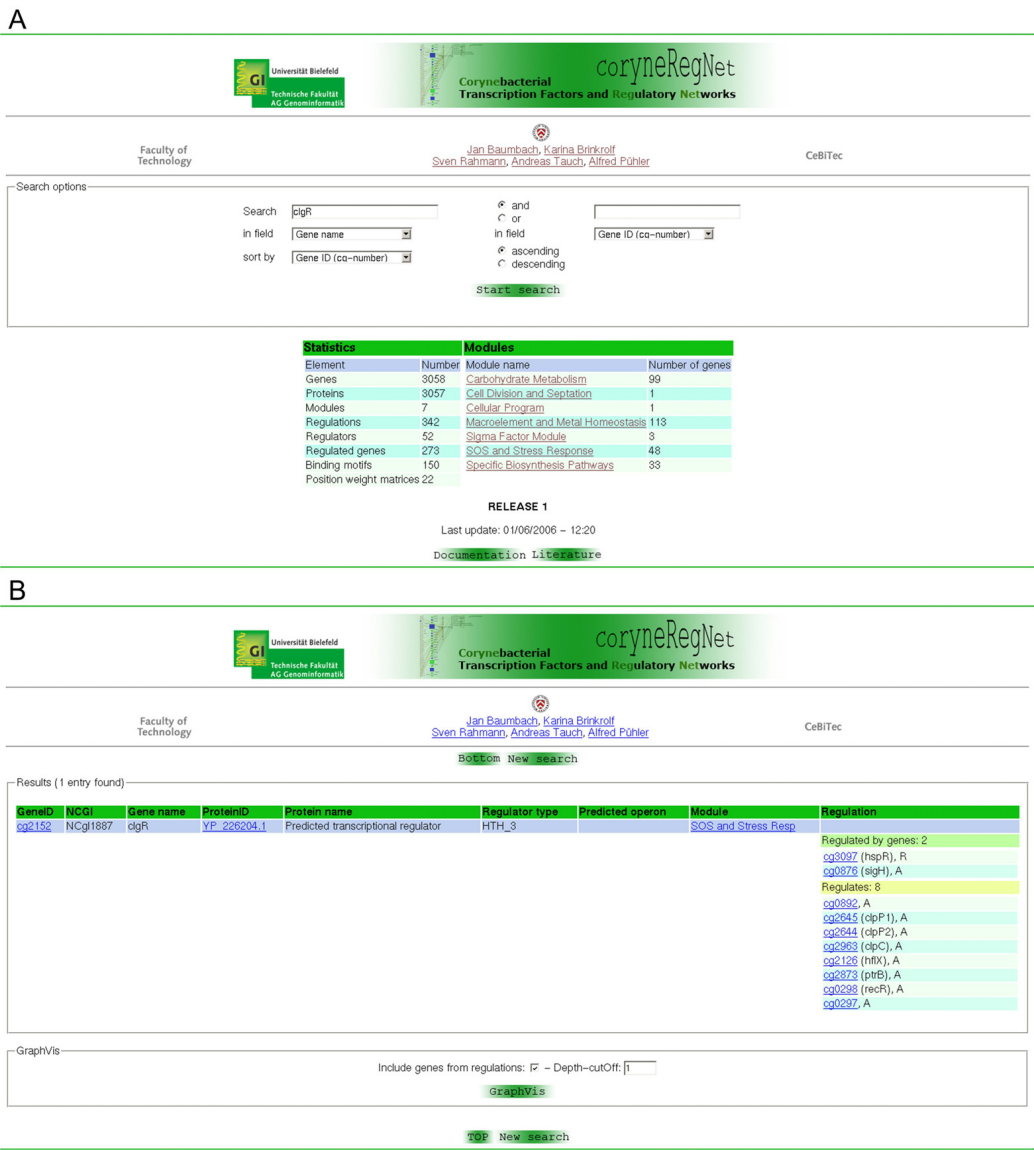
Web pages of CoryneRegNet. (A) Entry page of CoryneRegNet containing statistics and a search form. The database currently includes data on 52 transcriptional regulators exerting regulations on 273 genes. The regulators are classified into distinct physiological modules. The database can be searched by several criteria. (B) Exemplary results page representing relevant data of a searched item. Visualization of the respective transcriptional regulatory interactions by the GraphVis Java applet is applicable.

**Figure 5:**
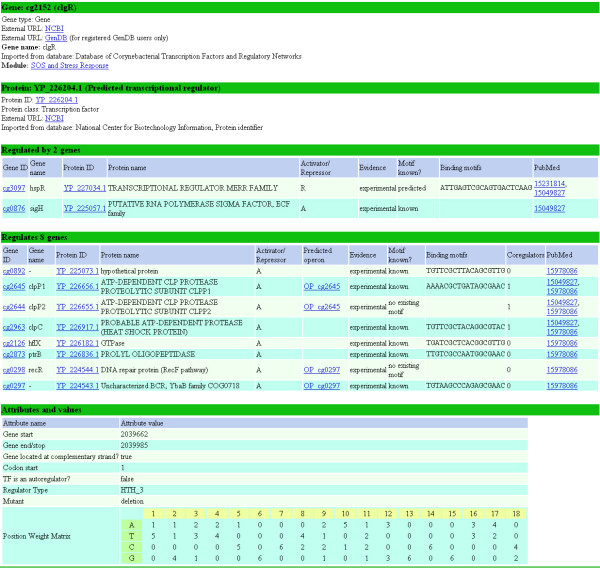
CoryneRegNet data page of a gene encoding a DNA-binding transcription factor. The page summarizes data relevant for the reconstruction of regulatory networks. It is linked to other databases such as NCBI Entrez, GenDB and PubMed. The example illustrates data for ClgR that is involved in stress response of *C. glutamicum *[39]. The DNA-binding motifs of ClgR are used to deduce a position weight matrix.

Web-based user interfaces to biological databases often support the following tasks: (i) *browsing *by listing or navigating through database entries, (ii) *searching *by identifying entries based on restrictions on the values of data fields within the database, (iii) *visualizing *by presenting a visual representation of the data, and (iv) *querying *by specifying a special search using a query building interface [31]. As well as other gene regulatory databases, such as PRODORIC [32,33], CoryneRegNet also emphasizes browsing, searching and visualizing. The entry page of CoryneRegNet shows a statistical summary of the data currently integrated into the database and provides the possibility to browse the functional modules of transcription factors (Figure 4A). Alternatively, the user can start searching the database using criteria that were obtained through a requirements analysis with potential users. The criteria are implemented following the typical search mask style (Figure 4A) of other gene regulatory databases, such as PRODORIC or TRANSFAC [32,34]. The search results are presented in a table-based style (Figure 4B) including gene and protein identifiers and names, the regulator type (if the specific protein is a transcription factor), the functional module the gene belongs to and the transcriptional regulations the gene is involved in. The user may acquire additional information on specific elements by clicking on them. A typical detailed view of data regarding a transcription factor gene is presented in Figure 5. It is possible to navigate to other entries of CoryneRegNet, to the genome annotation system GenDB [35] and to the NCBI Entrez Gene database [30] by following the respective links.

### Graphical visualization of regulatory interactions

**Figure 6:**
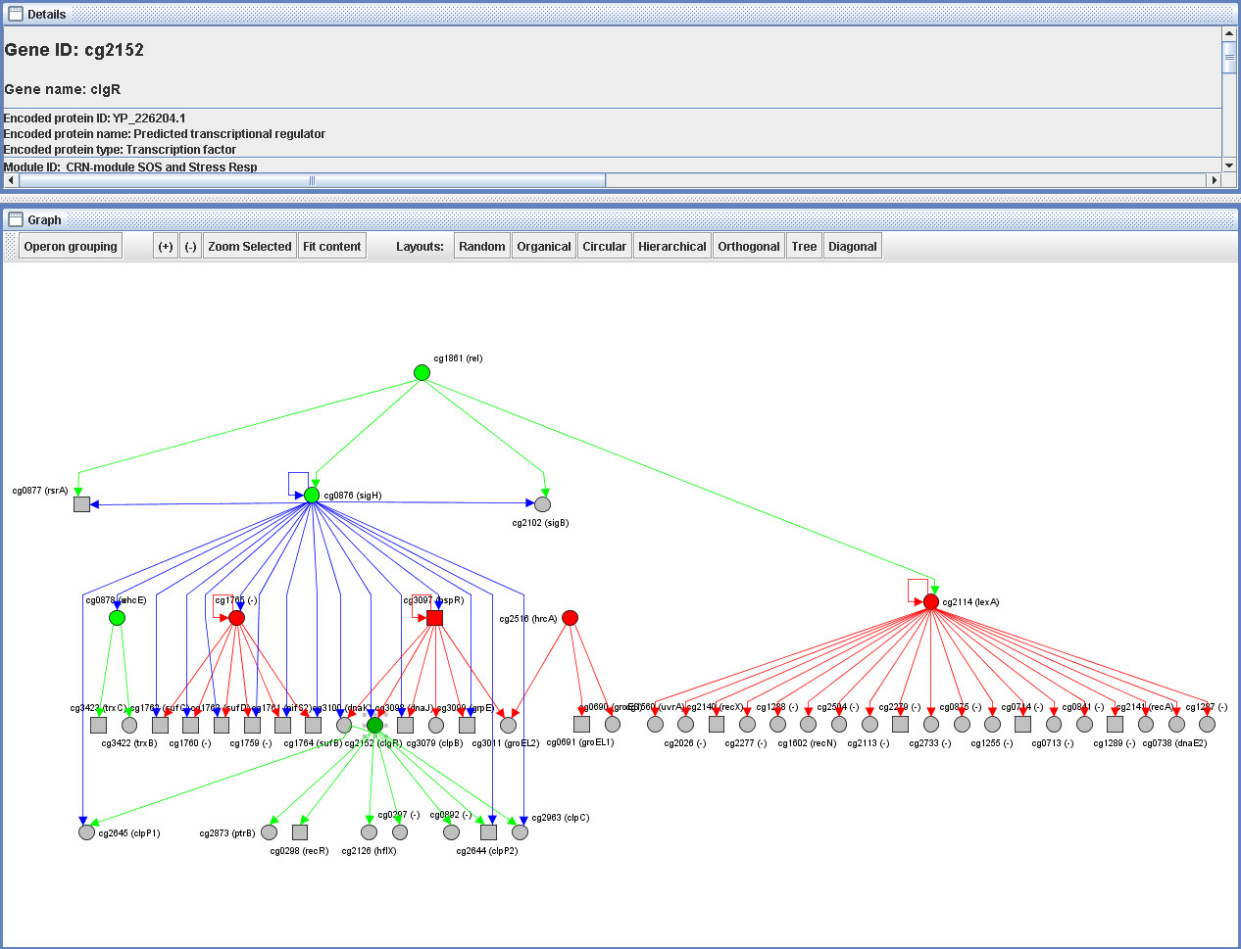
GraphVis Java applet showing the reconstruction of the SOS and stress response module of *C. glutamicum*. The graph was generated by means of the yFiles JAVA graph library using the hierarchical layout mode. Nodes represent genes included in this functional module. Color code: red node and line, repressor and repressing regulatory interaction; green node and line, activator and activating regulatory interaction; green node and blue line, sigma factor and sigma factor interaction; gray node, regulated target gene preceded by a transcription factor binding site; gray box, regulated target gene that is part of an operon and not preceded by a transcription factor binding site. The top level regulation of gene expression is indicated by the green node of the *rel *gene that is responsible for the cellular amount of ppGpp.

The user can visualize a transcriptional regulatory network at every navigation point using a result table or a detailed frame as starting point. The user has to define a graph depth cut-off and whether genes from hierarchical regulations should be included into the graph (Figure 4B). Graph construction starts with the selected set of genes, propagates through the regulatory network and adds more genes into the graph until the depth cut-off has been reached. The respective algorithm was implemented in PHP and generates a HTML file that in turn starts the Java applet GraphVis. Due to the security restrictions of the Java Virtual Machine, the whole graph is transferred by using named "PARAM tags" inside the "APPLET tag" before the GraphVis applet appears. Once the applet has been started, it is not able to "leave" the virtual machine. Accordingly, the whole graph along with all additional information on specific elements, for instance genes and proteins, is currently generated and transferred to the applet at start-up. Figure 6 shows an exemplary GraphVis Java applet window. The user obtains the same details on genes, proteins and regulatory interactions as in the browser-based view of CoryneRegNet. The main advantage is the graphical overview of the reconstructed regulatory network, where nodes in the graph represent genes and edges represent regulatory relationships. The user can zoom into the graph, layout the graph by using different styles, remove selected elements from the graph or retrieve detailed information on selected genes (Figure 6).

### Reconstruction of the SOS and stress response module

We used CoryneRegNet to reconstruct and visualize the transcriptional regulatory network of the SOS and stress response module of *C. glutamicum *(Figure 6). The module currently includes six DNA-binding transcription factors and 42 regulated genes. Since sigma factors play a key role in regulating gene expression when the cell is exposed to stress conditions and switches in part to the program "maintenance and survival" [21,36], the regulatory network is apparently linked to components of the sigma factor competition module. Thus, the reconstructed network reveals a hierarchical scheme also including the top level regulator ppGpp, synthesized by the Rel protein and influencing expression of the sigma factors SigH and SigB [21,22]. The reconstructed network allowed us to characterize the transcription factor module "SOS and stress response" in more detail: Several genes are under dual control by a DNA-binding transcription factor and by the alternative sigma factor SigH, whereas the *groEL2 *gene is co-regulated by two transcription factors. The network is additionally characterized by a number of autoregulatory loops (Figure 6) in which the transcription factor regulates its own expression. Regarding regulatory network motifs, the presence of feed-forward loops is apparent when considering the regulatory action on gene expression of both a transcriptional regulator (HspR or ClgR) and an alternative sigma factor (SigH). This is consistent with observations in *E. coli *that feed-forward loop motifs tend to be implemented within modules, whereas bi-fan motifs seem to be responsible for the connection between different physiological modules [5]. Two types of feed-forward loops are present in the reconstructed network of the SOS and stress response module, namely the coherent type 1 and the incoherent type 1 motif [37]. In a coherent type 1 feed-forward loop all the regulatory connetions are activating (SigH, ClgR, ClpP1-ClpP2), while in the incoherent type 1 motif one of the regulatory links represses the activity of the target node (SigH, HspR, DnaK). It is also apparent that the reconstructed regulatory network is composed of two distinct submodules reflecting different responses of the cell upon exposure to environmental stresses (Figure 6). The SOS response is induced by DNA damage and under control of the LexA protein, while the heat-shock and oxidative stress response is induced by denaturation and/or inactivation of proteins and is under SigH control [1]. Accordingly, the reconstruction and visualization of the SOS and stress response module of *C. glutamicum *by CoryneRegNet reflects the hierarchical and modular scheme of the cell's transcriptional regulatory system.

## Conclusion

With the recent progress made in large-scale postgenomic analysis of complete genome sequences a vast amount of novel data is becoming available. Comprehensive evaluation of postgenomic data asks for user-oriented databases supporting data management and data integration into existing knowledge. The CoryneRegNet database discloses detailed information on DNA-binding transcription factors, the key players in regulation of gene expression, and on transcriptional regulatory interactions of *C. glutamicum *deduced from literature-derived knowledge, computer predictions and global DNA microarray hybridization experiments. A web-based user interface provides access to the database content, allows various queries and supports the reconstruction and visualization of regulatory networks at different hierarchical levels. CoryneRegNet is moreover linked to the NCBI Entrez Gene database to provide direct access to corresponding genomic data. Although CoryneRegNet was developed as a data warehouse of transcriptional regulatory networks of *C. glutamicum*, its ontology-based design along with its programs and scripts is generally applicable to implement other species-specific databases. Consequently, CoryneRegNet is a versatile systems biology tool to support the large-scale analysis of transcriptional regulation of gene expression in microorganisms. The ultimate purpose of CoryneRegNet is to assist in reconstruction of transcriptional regulatory networks and to provide models that can be combined with metabolic networks of the cell to build integrated models including both cellular metabolism and transcriptional regulation. Since comparative computer analyses exploiting transcriptional regulatory data might be helpful to uncover hidden information on regulation of gene expression, transcriptional data of other sequenced corynebacterial species will be integrated into the next release of CoryneRegNet. For the future, we further plan to integrate existing and currently developing bioinformatics tools to perform for instance genome-wide searches for regulatory motifs specified by position weight matrices with sound statistical analysis [38] or to discover new potential motifs based on transcriptional profile analysis and comparative sequence analysis over several related genomes. We would also like to integrate algorithms and visualization techniques for comparing regulatory networks in several species. All of the above areas are active research fields with several new ideas being presented at every bioinformatics conference; therefore we are planning a flexible external tool plug-in concept for CoryneRegNet. Our long-term vision consists of CoryneRegNet proposing new regulatory hypotheses for wet-lab verification. While we expect that it will take some time for this vision to become reality, already now CoryneRegNet is a free open-source central repository and analysis tool for regulatory networks of microorganisms that is easy to extend because of its ontology-based design.

## Availability and requirements

The CoryneRegNet database is freely accessible through the website . Application of the yFiles JAVA graph library is restricted to academic users. Programs, scripts and information for setting up a species-specific database can be obtained from the authors upon request.

## Authors' contributions

JB designed and implemented CoryneRegNet and drafted the manuscript. KB developed the biological concept of CoryneRegNet, evaluated the data and participated in drafting the manuscript. LFC participated in data evaluation of the SOS and stress response module. SR participated in co-ordination and supervision and conceived of the design of CoryneRegNet. AT conceived of the study and participated in data evaluation and supervision. All authors read and approved the final manuscript.
